# Reversible control of magnetism in FeRh thin films

**DOI:** 10.1038/s41598-020-70899-x

**Published:** 2020-08-18

**Authors:** Dániel G. Merkel, Attila Lengyel, Dénes L. Nagy, Attila Németh, Zsolt E. Horváth, Csilla Bogdán, Maria A. Gracheva, Gergő Hegedűs, Szilárd Sajti, György Z. Radnóczi, Edit Szilágyi

**Affiliations:** 1 Wigner Research Centre for Physics, P.O.B. 49, 1525 Budapest, Hungary; 2 Centre for Energy Research, P.O.B. 49, 1525 Budapest, Hungary; 3 Institute of Chemistry, Eötvös Loránd University, Pázmány Péter sétány 1/A, 1117 Budapest, Hungary

**Keywords:** Nanoscale devices, Nanoscale materials, Nanoscale materials, Materials for devices, Information storage

## Abstract

The multilayer of approximate structure MgO(100)/[^n^Fe_51_Rh_49_(63 Å)/^57^Fe_51_Rh_49_(46 Å)]_10_ deposited at 200 °C is primarily of paramagnetic A1 phase and is fully converted to the magnetic B2 phase by annealing at 300 °C for 60 min. Subsequent irradiation by 120 keV Ne^+^ ions turns the thin film completely to the paramagnetic A1 phase. Repeated annealing at 300 °C for 60 min results in 100% magnetic B2 phase, i.e. a process that appears to be reversible at least twice. The A1 → B2 transformation takes place without any plane-perpendicular diffusion while Ne^+^ irradiation results in significant interlayer mixing.

## Introduction

Besides the traditional “faster–smaller–cheaper” directive, a new requirement has recently arisen for the newly developed devices: the energy efficiency. The electricity consumption of information technology is expected to reach 13% of the global utilization in the next decade^1,2^, of which nearly 50% is due to unwanted heat dissipation. Therefore, the study of novel materials and developing new operation principles for energy-saving applications in information technology are essential for supporting sustainable development. From this point of view, the fine control of magnetism is a great step towards reducing energy consumption of information storage by orders of magnitude^3–11^.

The Fe-Rh system is an excellent playground for developing energy-efficient magnetic devices^12,13^. The equilibrium phase diagram of solid Fe-Rh^14,15^ includes a great variety of phases of different magnetic behaviour. The disordered bcc A2 (α or δ, prototype W) phases are, depending on temperature, ferro- or paramagnetic (PM). The ordered bcc B2 (α’ or α’’, prototype CsCl) phases show ferro-, antiferro- or paramagnetism at different temperatures and concentrations. The disordered fcc A1 (γ, prototype Cu) phase is PM at all investigated temperatures.

The existence of a monoclinic antiferromagnetic (AFM) ground state was predicted by DFT calculations in the equiatomic FeRh alloy in 2016^16^. However, this phase could not be identified in thin films by Wolloch and co-workers^17^. Using nuclear resonant inelastic X-ray scattering (NRIXS), these latter authors analysed the lattice dynamical contribution to the phase stability in FeRh and demonstrated that the AFM ground state was stabilized by phonon softening.

With increasing temperature, the nearly equiatomic FeRh alloy of B2 structure undergoes a metamagnetic transition from the low-temperature AFM α’’ to the high-temperature ferromagnetic (FM) α’ phase close to room temperature (RT)^18–27^. In the AFM phase, Fe atoms carry a magnetic moment of 3.3 μ_B_ of alternating direction while the Rh atoms possess inconsiderable magnetic moment^22^. Conversely, in the FM phase, Fe and Rh atoms carry parallel moments of 3.2 μ_B_ and 0.9 μ_B_, respectively^28–30^. This magnetic transition is accompanied with a reduction of the resistivity and an ~ 0.6% isotropic strain of the crystal lattice^21,31^. By introducing strain in the FeRh crystal lattice, this phenomenon can be reversed and the magnetic transition can be triggered by deformation. This was demonstrated in 2014 by Cherifi and co-workers^12^ and investigated in more detail by Phillips and co-workers from the same group in 2015^32^ in a way that the deformation was induced by the piezoelectricity of BaTiO_3_ on which the FeRh thin layer was epitaxially evaporated. The electric-field switching of magnetism in this material proved to set a new record in the magnetoelectric coupling coefficient of ~ 1.6 × 10^–5^ sm^−1^ in multiferroic FeRh/BaTiO_3_ heterostructure^12^. The last six years saw an incredible growth in publications on developing devices based on multiferroic FeRh heterostructures. An alternative way of manipulating of the phase transition in ultrathin FeRh films by electric field through a combination of ionic liquid and oxide gating was demonstrated in 2016 by Jiang and co-workers so that the migration of oxygen ions was induced between the oxide gate and the FeRh layer^33^. The feasibility of building multiferroic FeRh/PMN-PT devices adjustable by only varying the amplitude of the applied electric field was shown in 2017 by Fina and co-workers^34^. As it has very recently been demonstrated by the same group, FeRh films preserving the temperature-dependent AFM–FM transition can be grown on a flexible metallic substrate coated with a textured MgO layer^35^, a fact making the FeRh/MgO interface especially relevant for large-scale applications.

Due to its marked influence on the magnetic properties, the B2 → A1 transition of the Fe-Rh system has been subject of extensive investigations. Since the A1 phase is metastable at RT, the traditional starting point of such studies was either annealing the bulk sample above 950 °C followed by a quenching procedure^36,37^ or plastic deformation by ball milling^37,38^ while the A1 → B2 transition was induced by a subsequent annealing at temperatures as low as 237 °C and slightly above^37,38^.

Recently, the manipulation of magnetic and structural properties of FeRh by ion irradiation has attracted great attention. Several experiments were carried out using different ions such as 1 MeV proton^39^, 10 MeV I^+^^39,40^, 30 keV Ga^+^^41^, 20 keV Ne^+^^42^ or 3.8 keV He^+^^43^ irradiation to tailor the FM–AFM transition of the B2 phase and/or to induce B2 → A1 structural transformation in FeRh thin films. Irradiation with protons and He^+^ ions as well as with 20 keV Ne^+^ ions resulted in a change of the total FM/AFM ratio of the B2 phase by decreasing the FM-AFM transition temperature close to RT^39,42^ or in a change of the depth profile of the magnetic properties of the B2 phase^43^. Conversely, bombardment with 10 MeV I^+^^39,40^ or 30 keV Ga^+^^41^ ions decreased or even diminished the B2 contribution in the originally mixed A1-B2 samples. By subsequent annealing at 500 °C after 10 MeV I^+^ irradiation, the original mixed A1-B2 state was retained^40^. Very recently, Eggert and co-workers have shown^44^ that low-fluency irradiation by 25 keV Ne^+^ ions ferromagnetism can be activated in an originally AFM B2 MgO(100)/FeRh film. Using slow positron annihilation, these authors evidenced additional open volumes in the film created by ion irradiation and established their depth profile. The profile was found to end before the MgO/FeRh interface. In this context, the authors emphasized the importance^45^ of minimizing the defect concentration in the substrate, a key point even if the depth resolution of the applied method is strongly limited by the broadness of the positron implantation depth distribution^46,47^.

The manipulation of magnetic properties by induced strain were also demonstrated in thin films by applying different (W, V) buffer layers^48,49^ or by varying the substrate temperature^50^. The effect of substrate-induced strain in case of MgO(100) substrate was also demonstrated^51^.

A key step towards applications in spintronic devices is building heterostructures with laterally alterable magnetic properties. In case of FeRh films, this may require developing techniques for a full and reversible control of the A1⟷B2 transformation. Indeed, this way B2 regions can be embedded in the PM A1 film separating them magnetically from each other and allowing for their further manipulation by focused ion or laser beams.

Here we report a study performed by conversion-electron Mössbauer spectroscopy (CEMS), high-angle X-ray diffraction (XRD), neutron reflectometry (NR), Rutherford backscattering spectrometry (RBS) and transmission electron microscopy (TEM) on an FeRh thin film deposited on MgO(100) substrate at 200 °C. We find that the main component of the as-deposited film is the PM A1 phase. A subsequent annealing at 300 °C turns the film completely into FM B2 phase. The PM A1 state is retained by 120 keV Ne^+^ ion irradiation. This state is turned to FM B2 again by annealing, etc., a process that can be repeated reversibly. Nevertheless, we stress that our present purpose is only to demonstrate the possibility of reversible control of magnetism in FeRh thin films without any immediate application in spintronic devices.

## Experimental results

### Sample history

[^n^FeRh/^57^FeRh]_10_ isotope-periodic multilayer was deposited on a 20 mm × 20 mm MgO(100) substrate at 200 °C using molecular beam epitaxy (MBE) technique (^n^Fe stands for Fe of natural isotopic abundance); the actual composition and thickness of the layers are given in chapter ‘Experimental methods’. TEM, RBS, CEMS, XRD and NR measurements were done on the as-deposited sample. Henceforth, the as-deposited state of the sample will be referred to as “*As-deposited*”.

The as-deposited sample was cut into several pieces. A piece of the sample was annealed at 300 °C for 60 min to see if and to which extent the ratio of magnetic and non-magnetic phases can be influenced by annealing. The status of the annealed sample was checked by CEMS, XRD and NR. The state of the sample after annealing at 300 °C for 60 min will be abbreviated as “*Annealed*”.

With the aim of further modifying magnetic properties, the *Annealed* sample was irradiated by 120 keV Ne^+^ ions, with a fluence of 1 × 10^16^ at/cm^2^. CEMS, XRD and NR experiments were performed after irradiation. The state of the sample after irradiation will be referred to as “*Irradiated*”.

Finally, the irradiated sample was annealed once more at 300 °C for 60 min. The sample was checked by CEMS and XRD. For this state of the sample the abbreviation “*Re-annealed*” will be used.

### Multilayer structure

**Figure 1:**
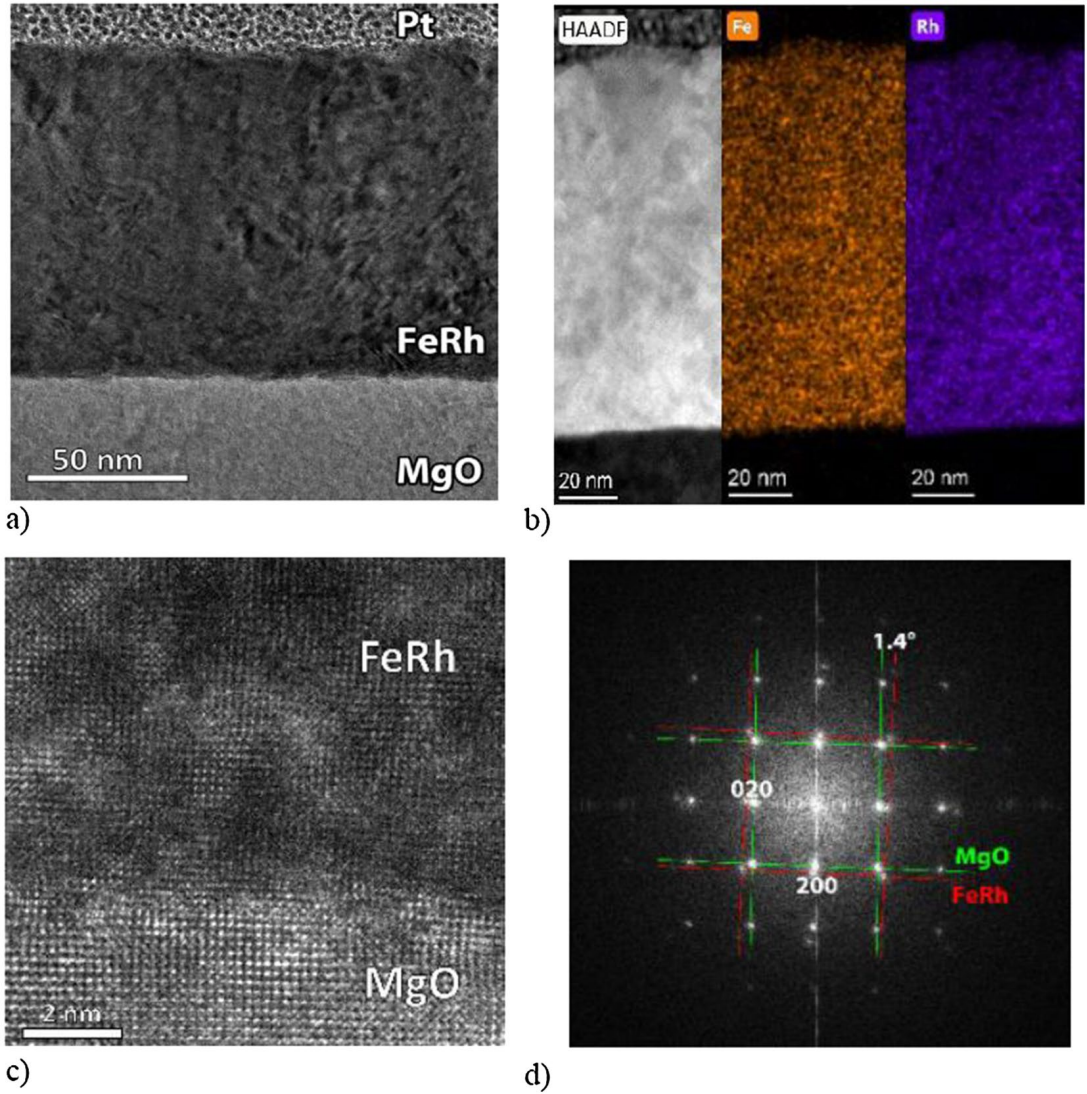
TEM overview image (**a**), high-angle annular dark-field STEM image, Fe and Rh EDX maps extracted from spectrum image (**b**) HRTEM image (**c**) and Fourier-transformed HRTEM lattice image for phase identification and epitaxial relationship (**d**) of the as-deposited sample.

The quality of the multilayer in the *As-deposited* state was primarily characterized by transmission electron microscopy (TEM) by imaging it both in TEM and scanning TEM (STEM) modes. To extract elemental maps, EDX spectrum images were recorded. In the respective Fig. 1a,b the TEM and high-angle annular dark-field (HAADF) STEM are shown. None of the images exhibits contrast between the adjacent ^57^FeRh and ^n^FeRh layers, i.e. no atomic concentration discrepancies within the multilayer are observed. In the upper interface, ~ 10 nm roughness can be seen. The Fe and Rh mappings reveal 44–48 at% Fe, 38–41 at% Rh and, due to FIB preparation, 11–12% Ga atomic concentration in the film.

High resolution TEM (HRTEM) image from the FeRh and MgO interface (Fig. 1c) indicates epitaxial growth of the FeRh layer. On the Fourier-transformed HRTEM lattice image (Fig. 1d) two, 1.4° misaligned, square lattices are visible. The smaller and the larger spatial frequencies correspond to MgO (200), 2.10(5) Å and A1 phase FeRh (200), 1.87(5) Å, respectively.

**Figure 2:**
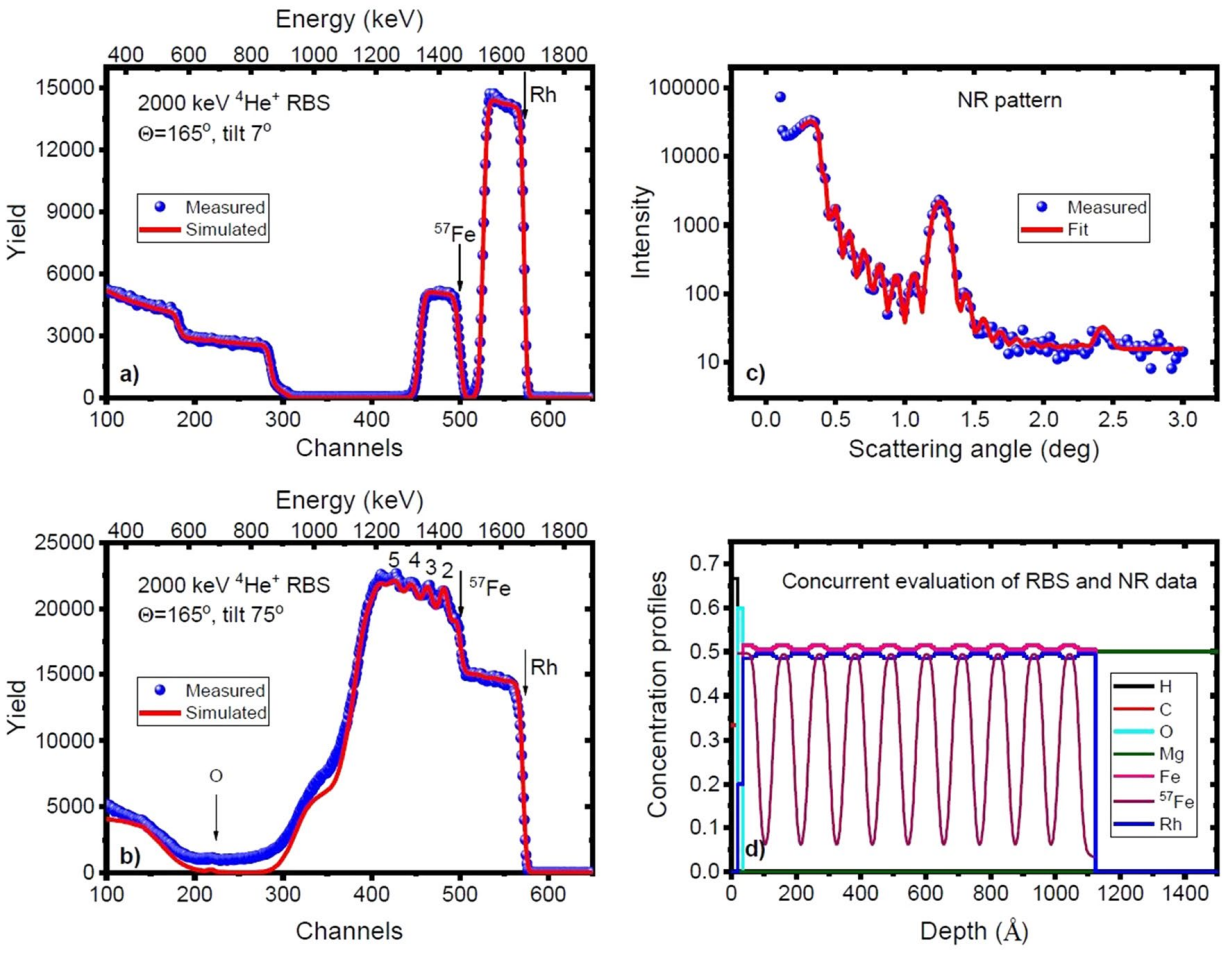
RBS spectra taken at tilt angles of 7° (**a**) and 75° (**b**) and neutron reflectivities (**c**) of the as-deposited sample. The corresponding element/isotope concentration depth profile from the concurrent evaluation of RBS and NR data is shown in panel (**d**).

The actual structure of the as-deposited sample was determined from a concurrent evaluation of RBS and NR data. Figure 2 shows the RBS spectrum at tilt angles 7° and 75° (a and b, resp.), the scattering-angle dependence of the neutron reflectivity (c) and the element/isotope concentration profile resulting from the concurrent evaluation of RBS and NR data (d).

The tilt angle 7° instead of perpendicular incidence was chosen to make sure to avoid channelling effects in the single-crystal substrate and epitaxial layers. The RBS spectra and the neutron reflectivities were calculated by the RBX code^52^ and the FitSuite program^53^, respectively. The structure was found to be MgO(100)/[^n^Fe_50.5_Rh_49.5_(63 Å)/^57^Fe_51.5_Rh_48.5_(46 Å)]_10_ with an error of the concentrations of ± 0.5.

The value of the slight difference in the concentrations of the ^n^FeRh and the ^57^FeRh layers was established from the little fringes in the Rh part of the tilt 75° RBS spectrum. The RBX code cannot account for plural scattering in the sample, a fact resulting in a significant difference between the low-energy parts of the measured and simulated spectra at tilt 75°. The well-defined Kiessig fringes in the neutron reflectivity are indicative of the good quality of the sample with flat surface while the Bragg peaks justify the isotopic periodicity, a fact also supported for the uppermost 5 bilayers by the Fe part of the tilt 75° RBS spectrum (Fig. 2b).

The final concentration profile shown in Fig. 2d was calculated by taking into account the isotope depth profile at the interfaces. In case of NR, the error-function interface profile was approximated with a step function, by generating a new (virtual) layer structure, characterized by *Dt* (squared diffusion length, *D* and *t* being the diffusion coefficient and the time of diffusion, respectively). In case of RBS, the diffusion profile was substituted by a mixed layer of width $\sqrt{Dt}$. From the evaluation, an initial mixing (diffusion length 7.6 Å) was concluded between the adjacent isotope layers.

Furthermore, the RBS spectrum shows the presence of a thin oxide layer at the surface of the sample. Tentatively associating it with FeRhO_3_, i.e. the Rh-substituted α-Fe_2_O_3_^54^ of calculated density 6.41 g/cm^3^^55^ and ilmenite crystal structure^56^, the uppermost ^57^Fe_51.5_Rh_48.5_(46 Å) layer was replaced by ^57^Fe_51.5_Rh_48.5_(35 Å)/ ^57^FeRhO_3_(16 Å).

### Formation, destruction and restoration of magnetic order

**Figure 3:**
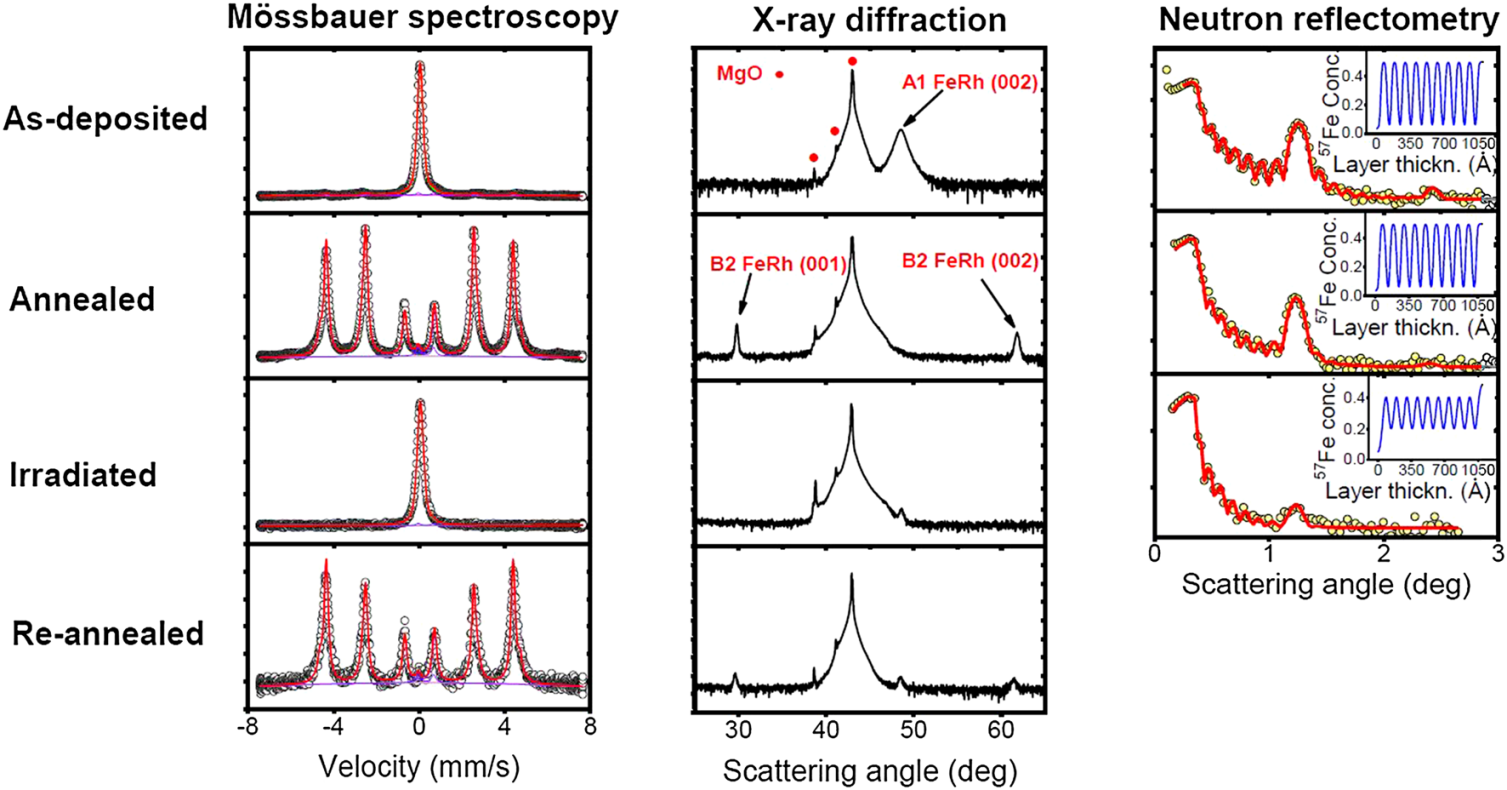
Conversion-electron Mössbauer spectroscopy (CEMS) spectra, high-angle X-ray diffraction (XRD), and unpolarized neutron reflectivity (NR) patterns recorded on the as-deposited MgO(100)/ [^n^FeRh/^57^FeRh] _10_ sample, after annealing for 60 min at 300 °C, after subsequent irradiation by 120 keV Ne^+^ ions, with a fluence of 1 × 10^16^ at/cm^2^ and after subsequent annealing for 60 min at 300 °C. Continuous curves in the CEMS and NR panels correspond to fits with parameters shown in Table 1 with the preceding two paragraphs and Table 3, respectively. In the inset of the neutron reflectometry graphs, the concentration profile of ^57^Fe is shown in the isotope layers. The ordinate of the CEMS panel is linear while those of the XRD and NR panels are logarithmic.

CEMS spectra, as well as XRD and NR patterns recorded on the sample in its *As-deposited*, *Annealed*, *Irradiated* and *Re-annealed* states in Fig. 3.

The strongest evidence supporting the main conclusion of the present paper, viz. the feasibility of full reversible control of magnetism in FeRh thin films comes from the CEMS experiments. CEMS spectra were evaluated with the MossWinn 4.0^57^ program. Since thickness effects are negligible in case of perpendicular-incidence CEMS^58^, as a rule, Lorentzian line shapes of natural linewidth 0.0960 mm/s calculated from the 99.2 ns half-life of the 14.414 keV isomeric state of ^57^Fe^59^ were used for fitting the subspectra. Conversely, the source spectrum was described by a pseudo-Voigt function accounting for resonant self-absorption in the source^60^ and instrumental vibrations. The Lorentzian and Gaussian widths of the pseudo-Voigt function (0.1376 mm/s and 0.0444 mm/s, resp.) were taken from the α-^57^Fe calibration spectra and were fixed when fitting CEMS spectra of the multilayers. Four main components, viz. two sextets, one singlet and a doublet could be identified in a simultaneous fit of all spectra. The sextets, the singlet and the doublet were assigned to the FM B2, the PM A1 phases and the surface oxide, respectively. In case of the sextet corresponding to Fe atoms in the B2 phase at their ordinary site, the scatter of the environment due to antisite Fe atom neighbours was described by a binomial distribution of the hyperfine field and the isomer shift. In the only case of the PM A1 singlet, also the multilayer spectrum was fitted with a pseudo-Voigt function of fixed Lorentzian width equal to the natural linewidth 0.0960 mm/s, however of free Gaussian width to account for minute unresolved distribution of the hyperfine fields.

Fitting the concentration of the antisite B2 Fe atoms, a common parameter for all spectra resulted in the composition of the ^57^FeRh layers of ^57^Fe_51.04(1)_Rh_48.96(1)_ (the numbers in bracket meaning the standard deviation in the last digits). This is in good agreement with the RBS/NR analysis of the *As-deposited* state. Further common parameters were the hyperfine magnetic field of the antisite B2 Fe atoms H^*^ = 39.2(4) T as well as their isomer shift δ^*^ = 0.273(2) mm/s. Likewise, the hyperfine magnetic field of the regular B2 Fe atoms was found to be *H*_0_ = 27.135(2) T and their isomer shift *δ*_0_ = 0.0214(2) mm/s. Also the quadrupole doublet of the surface oxide was evaluated in terms of common isomer shift *δ*_ox_ = 0.311(4) mm/s and quadrupole splitting *∆E*_ox_ = 0.715(7) mm/s. The percentile fraction of the surface oxide in the spectra was *r*_ox_ = 2.59(6) %; an overestimate of the atomic fraction of the oxide, due to the enhanced surface sensitivity of CEMS.

Parameters fitted separately in different states of the sample such as the relative fraction of the magnetic B2 phase within the multilayer ρ_B2_, the magnetic texture parameter of the B2 sextets *a* (i.e. the intensity of the second line of a sextet relative to the third), the isomer shift *δ*_A1_ of the A1 single line as well as its Gaussian broadening σ_A1_ are listed in Table 1. A comprehensive description and interpretation of the components as well as details of the analysis of the CEMS data is given in the Supplementary Information.

**Table 1:** Relative fraction of the magnetic B2 phase within the B2 and A1 phases *ρ*_B2_, magnetic texture parameter of the B2 sextets *a*, isomer shift *δ*_A1_ of the A1 resonance line and its Gaussian broadening *σ*_A1_ in the as-deposited MgO(100)/[^n^FeRh/^57^FeRh]_10_ sample and after the indicated treatment from conversion-electron Mössbauer spectroscopy (CEMS).

Parameter	As-deposited	Annealed	Irradiated	Re-annealed
*ρ*_B2_ (%)	6.0(3)	100.0(1)	0.8(7)	99.9(3)
*a*	3.247(7)	3.247(7)	2.32(2)	2.32(2)
*δ*_A1_ (mm/s)	0.0462(3)	0.0462(3)	0.0565(7)	0.0565(7)
*σ*_A1_ (mm/s)	0.163(1)	0.163(1)	0.207(2)	0.207(2)

The numbers in bracket mean the standard deviation in the last digits.

XRD peaks appearing at 2*ϴ* ≈ 29.7°, 43.0°, 48.6° and 61.7° can be assigned to B2 FeRh(001), MgO(002), A1 FeRh(002) and B2 FeRh(002), respectively^39,41^. Lattice parameters were calculated from the diffraction line positions using Bragg’s diffraction law. The approximate grain size of the B2 and A1 phases was calculated using the Scherrer Eq. ^61^. The extracted lattice parameters and grain sizes of both phases in various states of the sample are summarized in Table 2. Do note that in the *As-deposited* state only the A1 phase while in the *Annealed* and *Re-annealed* states only the B2 phase could be identified. Further properties of the of the A1 phase in the *Irradiated* and *Re-annealed* states will be described in chapter ‘Discussion’.

**Table 2:** Lattice parameters *d* and grain sizes *L* of the B2 and A1 phases in the as-deposited MgO(100)/ [^n^FeRh/^57^FeRh]_10_ sample and after the indicated treatment from high-angle X-ray diffraction (XRD).

Phase	Parameter	As-deposited	Annealed	Irradiated	Re-annealed
B2	*d* [Å]	N/A	3.008(1)	N/A	3.021(1)
	*L* [Å]	N/A	348(11)	N/A	230(10)
A1	*d* [Å]	3.761(1)	N/A	N/A	N/A
	*L* [Å]	78(4)	N/A	N/A	N/A

The numbers in bracket mean the standard deviation in the last digits. In case of the *Annealed* and *Re-annealed* states, the value of *L* was determined from the 2 *ϴ* ≈ 29.7° reflection, however, the analysis based on the 2 *ϴ* ≈ 61.7° reflection gives very similar results.

The CEMS and partly also the XRD data already at this stage clearly show that annealing results in formation of magnetic order in the almost completely non-magnetic as-deposited film; the same holds true for re-annealing the completely non-magnetic irradiated sample. The motivation of NR experiments was to check whether the primary annealing and the A1 → B2 transition caused was associated with any change of the isotope depth profile at the ^n^FeRh/^57^FeRh interfaces. The evaluation by the FitSuite code^53^ confirmed the first impression from panel NR of Fig. 3: no significant change in the parameters of the NR patterns was found. Conversely, Ne^+^ irradiation resulted in a substantial reduction of the isotope-periodic NR Bragg peak, i.e. considerable increase of the width of the ^n^FeRh/^57^FeRh interfaces. Since there was no reason to expect any further change in the NR parameters after re-annealing, this experiment was skipped.

Extracted values of the multilayer period length *d*_ML_ and the width of the mixed interface region expressed in terms of a hypothetical diffusion length $\sqrt{Dt}$ in different states of the sample are shown in Table 3.

**Table 3:** Multilayer period length *d*_ML_ and the width of the mixed interface region expressed in terms of a hypothetical diffusion length $\sqrt{Dt}$ of the as-deposited MgO(100)/ [^n^FeRh/^57^FeRh]_10_ sample and after the indicated treatment from unpolarized neutron reflectometry (NR).

Parameter	As-deposited	Annealed	Irradiated	Re-annealed
*d*_ML_ [Å]	109(1)	109(1)	109(1)	N/A
$\sqrt{Dt}$[Å]	7.6(2)	7.6(2)	16.7(5)	N/A

The numbers in bracket mean the standard deviation in the last digits.

**Figure 4:**
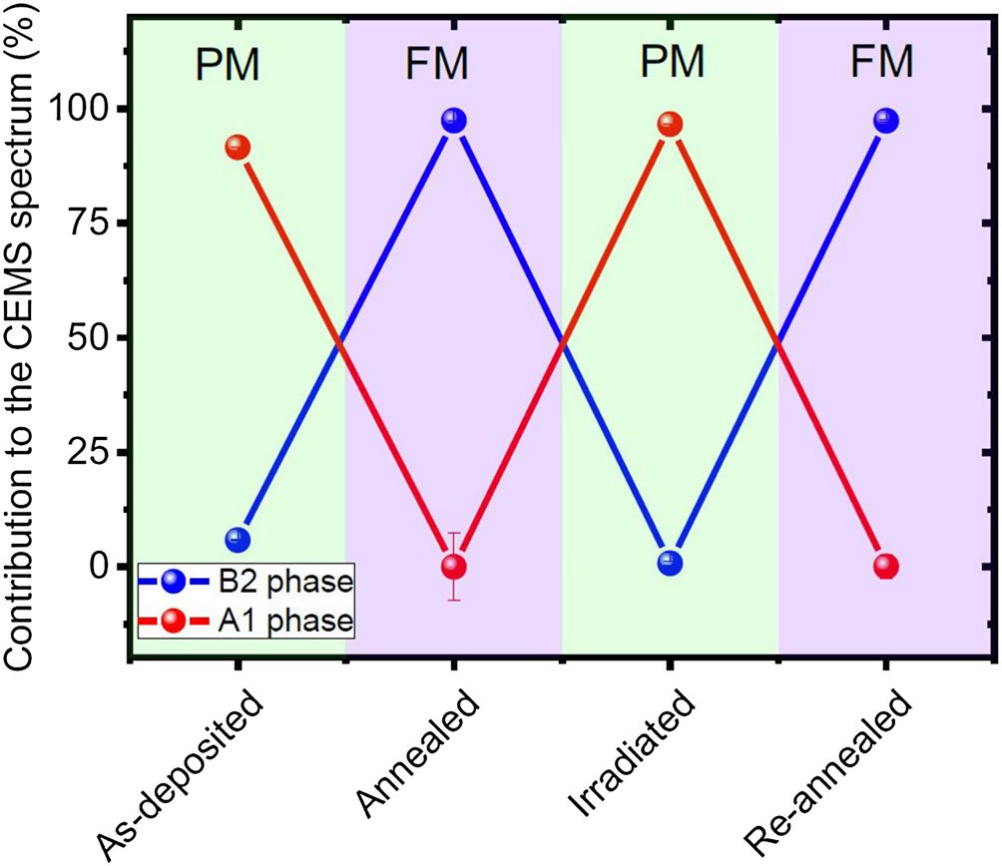
Contribution of the ferromagnetic B2 and paramagnetic A1 FeRh phases to the CEMS spectrum.

Figure 4 shows the evolution of the relative fractions of the ferromagnetic B2 and paramagnetic A1 phases without oxide contribution.

## Discussion

According to CEMS data, (Table 1) 94.0(3) % of the *As-deposited* sample (omitting the surface oxide) was in the A1 phase. This was also confirmed by XRD data (Table 2), the value of the lattice parameter *d* = 3.761(1) Å being close to the value *d* = 3.736 Å found for the A1 phase by Lommel & Kouvel^37^ in filings from a Fe_51_Rh_49_ ingot quenched from 975 °C, a temperature where the A1 was the equilibrium phase. The observed increase of the lattice parameter as compared to the bulk value indicates the presence of tensile stress in the *As-deposited* multilayer. The same authors reported that the slow A1 → B2 transformation induced by annealing started only at 237 °C and above. Therefore, the fact that the MBE deposition on the MgO substrate at *T* = 200 °C resulted dominantly in the A1 phase can be interpreted in terms of describing the deposition on a low-temperature (*T* ≲ 200 °C) substrate as quenching from high temperature so that atoms freeze in metastable positions. The presence of only the A1 phase was also evidenced by Witte et al.^49^ This latter author sputtered the FeRh layer on MgO/W-V substrate at ambient temperature. Indeed, as shown in Fig. 2 of the Supplementary Material of the paper by Witte et al.^48^, CEMS showed only the broad PM line (or unresolved quadrupole doublet) characteristic of the A1 phase. The isomer shift of *δ*_A1_ = 0.0462(3) mm/s proved to be slightly but significantly less than that suggested by Chao et al.^36^ (about 0.06 mm/s, cf. Figure 3 of the referenced paper) indicating that the A1 phase in the *As-deposited* state had a defect structure somewhat different from that of the A1 phase in quenched or ball-milled bulk samples.

Both CEMS (Table 1 and the two preceding paragraphs) and XRD (Table 2) indicated that after 60 min annealing at 300 °C (i.e. in the *Annealed* state) the multilayer was fully converted to the B2 (in fact, to the FM B2) phase. Indeed, apart from the minute contribution of the surface oxide layer, the CEMS spectrum (left panel of Fig. 3) of the *Annealed* sample consisted only of magnetic sextets characteristic of the B2 phase. The respective fit values of the hyperfine magnetic field and the isomer shift of the antisite B2 Fe atoms (Fe II in Shirane et al.’s^62^ notation) H^*^ = 39.2(4) T and δ^*^ = 0.273(2) mm/s, are in fair agreement with those found by Shirane et al.^28,62^ H^*^ = 38.2 T and δ^*^ = 0.32(2) mm/s for the composition Fe_52_Rh_48_. The latter value was derived, with an admittedly limited accuracy, from the graphical evaluation of figure 5 of the paper of Shirane et al.^62^ and the isomer shift − 0.155(2) mm/s of the ^57^Co(Cr) source^63^. Likewise, only B2 reflections in the XRD pattern (middle panel of Fig. 3) could be identified in the *Annealed* state. The lattice parameter of the B2 phase in the *Annealed* state *d* = 3.008(1) Å (cf. Table 2) is in fair agreement with previous works (3.020 Å^64^, 3.012 Å^65^ and 3.018 Å^66^), indicating that the transformation released most of the mechanical stress in the system. The annealing process increased the grain size *L* of the multilayer by about a factor 4.4 (78(4) Å for the A1 phase in the *As-deposited* state as compared with 348(11) Å for the B2 phase in the *Annealed* state, cf. Table 2).

Ion irradiation has two important effects on the multilayers. First, the so-called ion-beam mixing due to collision cascades. Second, the energy deposition of the ions that can be considered as a heat treatment of the material including melting and re-solidification and/or phase transition along the track of the ions the radii of which can be characterized by the stopping power of the ions and the material properties those falling typically to the region of 2–10 nm. The 120 keV energy of the Ne^+^ ions used for irradiation was chosen on the basis of detailed collision calculations using the SRIM code^67,68^. It appeared that, in average, each ion induces 1582 vacancies during cascade events and stopping at the substrate at range 1,369 Å with 926 Å straggling. This means that ion-beam mixing affected the whole multilayer, however most of the vacancies were created close to the FeRh/MgO interface and also Ne atoms stopped there forming bubbles and voids. Therefore, the FeRh/MgO interface may have a behaviour different from that of the FeRh multilayer.

The CEMS spectrum clearly shows that the *Irradiated* sample was practically fully (actually to 99.2(7)%, cf. Table 1) converted to the PM A1 phase. In accordance with this, the XRD pattern contains no B2 reflections. The origin of the A1 reflection observed at 2*ϴ* ≈ 48.6° in the *Irradiated* state is still somewhat perplexing. Indeed, the intensity of this reflection is only a negligible fraction of that of the same A1 reflection in the *As-deposited* state, in spite of the fact that the A1 contribution in the latter case was even less, viz. only 94.0(3)%. The obvious explanation of this apparent contradiction is that the main XRD contribution from the A1 phase in the *Irradiated* state was X-ray-amorphous, i.e. had a grain size of about 20 Å or less smearing out the sharp reflection. Do note that the formation of A1 grains of a few nm size from the B2 phase was also observed in FeRh alloys after high-speed deformation^69^. The minute sharp A1 reflection corresponding to the lattice parameter *d* = 3.753(1) Å and grain size *L* = 111(6) Å probably comes from the FeRh/MgO interface where, due to the vacancies and voids^44^ a small quantity of bigger grains of the A1 phase was formed. In view of the about 50 nm information depth of CEMS^58^, the FeRh/MgO interface is practically not seen by the latter method. It seems that irradiation with 120 keV Ne^+^ ions not only resulted in a practically full B2 → A1 transformation but also in comminuting most of the B2 grains of size *L* = 348(11) Å (cf. Table 2) to by an order of magnitude smaller ones.

Re-annealing resulted in a full conversion of the multilayer to the magnetic B2 phase. Indeed, CEMS evidenced 99.9(3) % B2 phase in the *Re-annealed* state (cf. Table 1). The grain size *L* = 230(10) Å (cf. Table 2) of the B2 phase was again larger than that of the mother A1 phase in the *Irradiated* state, however much less than the B2 grain size *L* = 348(11) Å in the *Annealed* state (cf. Table 2). It would be tempting to argue that the decrease of the B2 grain size in the *Re-annealed* state is connected with the fact that those grains were formed from A1 grains of the *Irradiated* state that were much smaller than the A1 grains in the *As-deposited* state (*L* = 78(4) Å, cf. Table 2). However, it is rather believed that the B2 grain size is determined by thermodynamic parameters controlling the nucleation of the A1 → B2 transition that may be different for the *As-deposited* → *Annealed* and the *Irradiated* → *Re-annealed* transformations. The value of the lattice parameter measured in the *Re-annealed* state *d* = 3.021(1) Å (cf. Table 2) is in an excellent agreement with literature data (3.020 Å^64^, 3.012 Å^65^ and 3.018 Å^66^). This shows that re-annealing practically fully removed all lattice defects created by Ne^+^ irradiation.

According to Table 1, CEMS showed that the relative fraction of the A1 phase in the *As-prepared* state within the B2 and A1 phases was 94.0(3) %, in accord with the only significant contribution to the XRD pattern in the same state, viz. the strong and broad A1 FeRh(002) reflection (cf. the XRD panel in Fig. 3). In the *Irradiated* and the *Re-annealed* states, CEMS indicated an A1 contribution of 99.2(7) % and 0.1(3) %, respectively. Nevertheless, as evidenced by the XRD pattern, some part of the sample in the *Re-annealed* state remained in the A1 state. As outlined in chapter ‘Experimental methods’, XRD is, as a rule, not suitable for quantitative phase analysis. Especially in case of epitaxial or textured layers, the intensity of an XRD reflection very much depends on the exact orientation of the sample, the degree of texture, the relative orientation of the sample surface and the preferred crystallite orientation as well as other parameters. Besides, X-ray amorphous regions do not contribute to the XRD pattern. At least for the Fe-containing phases, the only reliable quantitative information can be gained from CEMS (cf. Table 1 and the Supplementary Information).

The model of the irradiation-induced grain comminution is strongly supported by comparing the magnetic texture of the B2-phase multilayer in the *Annealed* and *Re-annealed* states. The magnetic texture parameter *a* is extracted from the line intensities of the magnetic sextets by describing them as 3:*a*:1:1:*a*:3. If the ferromagnetic grains are large, the shape anisotropy of the film aligns their magnetization (and the hyperfine magnetic field) parallel to the plane corresponding to *a* = 4. The magnetization of small grains is randomly oriented resulting in *a* = 2. The values *a* = 3.247(7) and *a* = 2.32(2) found in the *Annealed* and the *Re-annealed* state, respectively confirm that the distribution of the magnetization changed by irradiation from almost plane-parallel to nearly random.

In accordance with expectations, NR showed the same multilayer period length *d*_ML_ = 109(1) nm in the *As-deposited*, *Annealed* and *Irradiated* states. Quite surprisingly, exactly the same values of the hypothetical diffusion length $\sqrt{Dt}$ = 7.6(2) Å were found in the *As-deposited* and *Annealed* states. As anticipated, the 120 keV Ne^+^ irradiation resulted in a significant interlayer mixing leading to $\sqrt{Dt}$ = 16.7(5) Å in the *Irradiated* state (cf. Table 3).

In the work of Navarro et al.^38^, the activation-energy-controlled polymorphous transformation from the disordered A1 to the ordered B2 phase of FeRh was investigated. These authors obtained *Q* = 194 kJ mol^−1^ for the activation energy along with *D*_0_ = 0.08 cm^2^s^−1^ for pre-exponential factor and, accordingly, expected that at annealing at *T* = 327 °C for 60 s would result in $\sqrt{Dt}$ ≈ 72 Å diffusion length. Quite the contrary, no diffusion across the isotope layers (i.e. perpendicular to the film plane) connected with the A1 → B2 transformation was observed in the present study in spite of the dramatic changes between the *As-deposited* and *Annealed* states witnessed by CEMS and XRD. Consequently, the transformation occurred either by lateral diffusion or by single-pair exchange between neighbouring atoms. The origin of this peculiar behaviour of the A1 → B2 transformation is not fully understood at this stage but it most likely comes from the properties of the A1 phase in the *As-deposited* state, as a consequence of the preparation method. Indeed, in the XRD spectra the A1 phase only with the (002), while the B2 phase with (001) (002) planes appear, therefore, if the diffusion is anisotropic (akin to the case of L1_0_ FePt^70^), it is possible that, despite the enhanced lateral atomic diffusion, only minute atomic movement through in the plane-perpendicular direction occurred. Moreover, the small grain size and presence of tensile stress suggest that the *As-deposited* A1 structure was in a biased state energetically favouring the A1 → B2 transformation with very short atomic movements.

## Conclusions

In conclusion, we have shown that the thin film of approximate composition Fe_51_Rh_49_ deposited by molecular beam epitaxy on MgO(001) substrate at 200 °C contains primarily the paramagnetic A1 phase with a minute contribution from the magnetic B2 phase. The as-deposited state is fully converted to the magnetic B2 phase by annealing at 300 °C for 60 min. Subsequent irradiation by 120 keV Ne^+^ ions with a fluence of 1 × 10^16^ at/cm^2^ turns the thin film completely to the paramagnetic A1 phase. Repeated annealing at 300 °C for 60 min results in 100% magnetic B2 phase, a process that seems to be suitable for being repeated reversibly several times.

Neutron reflectometry performed on isotope-periodic multilayer evidenced that the conversion of the as-deposited, mainly A1 phase containing film to the B2 phase by annealing takes place without any plane-perpendicular diffusion, i.e. either by lateral diffusion or by single-pair exchange between neighbouring atoms. Conversely, Ne^+^ irradiation results in significant interlayer mixing.

These results open the way to fabricating nearly equiatomic FeRh thin films of designed lateral magnetic/non-magnetic structure for future applications in spintronic devices. Possible approaches may be both lithographic techniques and heating by focused laser-beams. These applications are, however, beyond the scope of the present paper.

## Experimental methods

The ^n^FeRh/^57^FeRh bilayer sequence was prepared by co-evaporation using electron gun for ^n^Fe (0.0248 Å/s) and Rh (0.0440 Å/s), and effusion cell for ^57^Fe (0.0248 Å/s) evaporation (isotopic enrichment 95%). To achieve better homogeneity, the substrate was rotated continuously during sample preparation. The substrate temperature was held at 200 °C and the pressure during evaporation never exceeded 1.2 × 10^–8^ mbar. Prior to evaporation, the substrate was cleaned in ultrasonic ethanol bath, then it was baked out at 630 °C for 30 min under ultrahigh vacuum (UHV) conditions. The quality of the epitaxial growth was monitored by an in-situ reflection high-energy electron diffraction (RHEED) facility during deposition, the recorded image is shown in the Supplementary Information.

The sample in the *As-deposited* state was imaged in a Themis transmission electron microscope by Thermo Fischer Scientific operated in TEM and STEM modes with 200 kV accelerating voltage and low-background double-tilt sample holder for energy-dispersive X-ray spectroscopy (EDX) analysis. EDX spectrum images were recorded for extraction of elemental maps. Focused ion beam (FIB) sections were prepared from the *As-deposited* sample. First, a thin Pt layer was deposited by electron-induced deposition, followed by a 2 µm thick layer using ion-induced deposition. Then, thin sections were cut using a 30 keV Ga beam. The final cleaning step was carried out at 5 kV, in order to keep the thermal load on the sample minimal, however, this way, the sample was contaminated by a small amount of Ga. Conventional Ar ion milling was forborne in order to evade the relatively high heating power of the Ar ion beam comparable with the heat treatments of the samples.

The RBS measurements in the *As-deposited* state were performed using 2 MeV ^4^He^+^ ion beam obtained from the 5 MV Van de Graaff accelerator of Wigner RCP. The beam was collimated to the necessary dimensions of 0.5 × 0.5 mm^2^ with two sets of four-sector slits and the measurements were performed with an ORTEC detector of a solid angle of 4.657 msr. The dose of the measurements was 20 μC. The ion current of typically 8 nA was measured by a transmission Faraday cup^71^. To reduce the surface contamination, liquid N_2_ trap was used; the pressure in the scattering chamber was about 1.5 × 10^–6^ mbar during the experiments.

The irradiation of the sample was carried out in the 450 kV heavy-ion cascade implanter of Wigner RCP. The pressure in the irradiation chamber was 3 × 10^–7^ mbar. The ion beam of about 1 mm diameter was *x*–*y* scanned across the full sample surface in order to achieve good homogeneity of irradiation within the exposed area. The current density for the scanning beam was 750 nA/cm^2^ ensuring that the temperature of the multilayer during irradiation was less than 50 °C. An important reason for selecting Ne^+^ ions for the irradiation was that Ne is a noble gas and, therefore, it is not expected to form any chemical phases in the sample.

Heat treatment of both the *As-deposited* sample and the sample in its *Irradiated* state leading to the *Annealed* and *Re-annealed* states, respectively, were performed under high vacuum of about of 10^–7^ mbar.

Mössbauer spectroscopy and, accordingly, CEMS is an extremely local method in the sense that it reflects hyperfine interactions within no more than a few coordination shells of the resonant nucleus. It is equally sensitive to any species irrespective whether they are embedded in single crystals, polycrystals or grains of any size. Nevertheless, a CEMS experiment gives a laterally averaged information on the whole sample. Furthermore, the mean free path of conversion electrons in similar systems is about 50 nm^58^ and, therefore, regions of a thin film beyond this value contribute only negligibly to the CEMS spectrum.

CEMS experiments were done using a conventional WissEl/DMSPCA Mössbauer spectrometer operated in constant-acceleration or sinusoidal drive mode at drive frequency 16 Hz. The actual activities of the originally 25 mCi or 50 mCi ^57^Co(Rh) single-line Mössbauer sources were in the range 8 mCi to 23 mCi at the time of the measurements. Resonant conversion electrons were detected with a homemade gas-flow single-wire proportional counter of 1 mm distance between sample and anode wire and working with a mixture of 96% He and 4% CH_4_ gas at various bias voltages between 875 V and 1,055 V. The distance between source and sample was 53 mm. The size of the samples was 5 mm × 5 mm, 5 mm × 10 mm or 10 mm × 10 mm. Throughout this paper, values of the isomer shift are given with respect to α-Fe at room temperature.

In contrast to CEMS, XRD is a non-local method yielding structural information on a macroscopic region of the thin film sample at any depth of several μm (i.e. much beyond the total thickness of the sample of the present study). It is indispensable for identifying all kinds of phases occurring in a sample, however its applicability for quantitative phase analysis can be very limited. Especially in case of epitaxial or textured layers, the intensity of an XRD reflection very much depends on the exact orientation of the sample, the degree of texture, the relative orientation of the sample surface and the preferred crystallite orientation as well as other parameters. Moreover, it is very sensitive to the grain size so that X-ray amorphous regions will not contribute to the XRD pattern at all.

XRD patterns were taken by a Bruker D8 Discover type diffractometer using Cu Kα radiation (*λ* = 1.5418 Å). To decrease beam divergence and to improve the parallelism of the beam, 0.6 mm slits were used at the source and the detector and a 90° rotated Soller slit was installed between the sample and the detector-side slit. For a better signal-to-noise ratio, a secondary monochromator was used at the detector side.

Unpolarized NR was performed at the GINA neutron reflectometer at the Budapest Neutron Centre^72,73^. The 4.61 Å wavelength monochromatic beam was provided by a pyrolytic graphite monochromator and the higher harmonics were removed by using liquid nitrogen cooled Be-filter. The neutron beam was collimated by two slits down to 0.6 mm size in the scattering direction. The specularly reflected neutrons were detected by a position-sensitive detector.

CEMS, XRD, NR, RBS and TEM experiments were done at RT.

## Supplementary information

Supplementary information

## Data Availability

The data that support the findings of this study are available from the corresponding author upon request.

## References

[1] Avgerinou M, Bertoldi P, Castellazzi L (2017). Trends in data centre energy consumption under the European code of conduct for data centre energy efficiency. Energies.

[2] Feng Z, Yan H, Liu Z (2019). Memristors: A spin–orbit-torque memristive device. Adv. Electron. Mater..

[3] Ohno H (2000). Electric-field control of ferromagnetism. Nature.

[4] Ramesh R, Spaldin NA (2007). Multiferroics: Progress and prospects in thin films. Nat. Mater..

[5] Maruyama T (2009). Large voltage-induced magnetic anisotropy change in a few atomic layers of iron. Nat. Nanotechnol..

[6] Spaldin NA, Cheong S, Ramesh R (2010). Multiferroics: Past, present, and future. Phys. Today.

[7] Vaz CAF (2012). Electric field control of magnetism in multiferroic heterostructures. J. Phys. Condens. Matter..

[8] Heron JT, Schlom DG, Ramesh R (2014). Electric field control of magnetism using BiFeO_3_-based heterostructures. Appl. Phys. Rev..

[9] Bi C (2014). Reversible control of co magnetism by voltage-induced oxidation. Phys. Rev. Lett..

[10] Matsukura F, Tokura V, Ohno H (2015). Control of magnetism by electric fields. Nat. Nanotechnol..

[11] Song C, Cui B, Li F, Zhou X, Pan F (2017). Recent progress in voltage control of magnetism: Materials, mechanisms, and performance. Prog. Mater. Sci..

[12] Cherifi RO (2014). Electric-field control of magnetic order above room temperature. Nat. Mater..

[13] Fina I (2018). Reversible and magnetically unassisted voltage-driven switching of magnetization in FeRh/PMN-PT. Appl. Phys. Lett..

[14] Swartzendruber LJ (1984). Bull. Alloy Phase Diagrams.

[15] Watts, G. R. *Gmelin Handbook Rh Suppl.* Vol. A 1, 232, (Springer, Berlin, 1991).

[16] Aschauer U, Braddell R, Brechbühl SA, Derlet PM, Spaldin NA (2016). Strain-induced structural instability in FeRh. Phys. Rev. B.

[17] Wolloch M (2016). Impact of lattice dynamics on the phase stability of metamagnetic FeRh: Bulk and thin films. Phys. Rev. B.

[18] Fallot M (1938). Les alliages du fer avec les métaux de la famille du platine. Ann. Phys..

[19] Fallot M, Hocart R (1939). Sur l'apparition du ferromagnétisme par élévation de température dans des alliages de fer et de rhodium. Rev. Sci..

[20] Muldawer L, de Bergevin F (1961). Antiferromagnetic-ferromagnetic transformation in FeRh. J. Chem. Phys..

[21] Kouvel JS, Hartelius CC (1962). Anomalous magnetic moments and transformations in the ordered alloy FeRh. J. Appl. Phys..

[22] Bertaut F, de Bergevin F, Roult G (1963). Magnetisme-etude par diffraction neutronique de Fe_0,47_Rh_0,53_. Compt. Rend..

[23] Lommel JM (1966). Magnetic and electrical properties of FeRh thin films. J. Appl. Phys..

[24] Heeger AJ (1970). Pressure dependence of the FeRh first-order phase transition. J. Appl. Phys..

[25] Schinkel CJ, Hartog R, Hochstenbach FHAM (1974). On the magnetic and electrical properties of nearly equiatomic ordered FeRh alloys. J. Phys. F: Met. Phys..

[26] Amirov AA (2019). Magnetic phase transition and magnetoelectric coupling in FeRh/PZT film composite. J. Magn. Magn. Mat..

[27] Warren JL, Barton CW, Bull C, Thomson T (2020). Topography dependence of the metamagnetic phase transition in FeRh thin films. Sci. Rep..

[28] Shirane G, Chen CW, Flinn PA, Nathans R (1963). Hyperfine fields and magnetic moments in the Fe–Rh system. J. Appl. Phys..

[29] Moruzzi VL, Marcus PM (1992). Antiferromagnetic-ferromagnetic transition in FeRh. Phys. Rev. B.

[30] Arregi JA (2018). Magnetization reversal and confinement effects across the metamagnetic phase transition in mesoscale FeRh structures. J. Phys. D: Appl. Phys..

[31] Lewis LH, Marrows CH, Langridge S (2016). Coupled magnetic, structural, and electronic phase transitions in FeRh. J. Phys. D: Appl. Phys..

[32] Phillips LC (2015). Local electrical control of magnetic order and orientation by ferroelastic domain arrangements just above room temperature. Sci. Rep..

[33] Jiang M (2016). Electrochemical control of the phase transition of ultrathin FeRh films. Appl. Phys. Lett..

[34] Fina I (2017). Electric-field-adjustable time-dependent magnetoelectric response in martensitic FeRh alloy. ACS Appl. Mater. Interfaces.

[35] Fina I (2020). Flexible antiferromagnetic FeRh tapes as memory elements. ACS Appl. Mater. Interfaces.

[36] Chao CC, Duwez P, Tsuei CC (1971). Metastable fcc Fe-Rh alloys and the Fe-Rh phase diagram. J. Appl. Phys..

[37] Lommel JM, Kouvel JS (1967). Effects of mechanical and thermal treatment on the structure and magnetic transitions in FeRh. J. Appl. Phys..

[38] Navarro E, Yavari AR, Hemando A, Marquina C, Ibarra MR (1996). Enthalpies of B2 antiferro-ferromagnetic and metastable fcc-B2 transformations in FeRh. Sol. State Commun..

[39] Fujita N (2010). Magnetic states controlled by energetic ion irradiation in FeRh thin films. J. Appl. Phys..

[40] Kosugi S (2011). Effect of high temperature annealing on ferromagnetism induced by energetic ion irradiation in FeRh alloy. Nucl. Instr. Methods B.

[41] Aikoh K, Kosugi S, Matsui T, Iwase A (2011). Quantitative control of magnetic ordering in FeRh thin films using 30 keV Ga ion irradiation from a focused ion beam system. J. Appl. Phys..

[42] Heidarian A (2015). Tuning the antiferromagnetic to ferromagnetic phase transition in FeRh thin films by means of low-energy/low fluence ion irradiation. Nucl. Instr. Methods B.

[43] Bennett SP (2018). Magnetic order multilayering in FeRh thin films by He-Ion irradiation. Mater. Res. Lett..

[44] Eggert B (2020). Magnetic response of FeRh to static and dynamic disorder. RSC Adv..

[45] Ricci D (2003). Paramagnetic defect centers at the MgO surface an alternative model to oxygen vacancies. J. Am. Chem. Soc..

[46] Dryzek J, Horodek P (2008). GEANT4 simulation of slow positron beam implantation profiles. Nucl. Instr. Methods B.

[47] Li C (2019). Implantation profiles and depth distribution of slow positron beam simulated by Geant4 toolkit. Phys. Scr..

[48] Witte R (2016). Tailoring magnetic frustration in strained epitaxial FeRh films. Phys. Rev. B.

[49] Witte R (2019). Epitaxial strain adaptation in chemically disordered FeRh thin films. Phys. Rev. B.

[50] Lu W, Huang P, Li K, Yan B (2013). Effect of substrate temperature on the crystallographic structure and first-order magnetic phase transition of FeRh thin films. J. Mater. Res..

[51] Barton CW (2017). Substrate induced strain field in FeRh epilayers grown on single crystal MgO (001) substrates. Sci. Rep..

[52] Kótai E (1994). Computer methods for analysis and simulation of RBS and ERDA spectra. Nucl. Instr. Methods B.

[53] Sajti, Sz. & Spiering, H. *FitSuite*. https://www.fs.kfki.hu.

[54] Seki, M. Bandgap-Engineered Iron Oxides for Solar Energy Harvesting, in: *Iron Ores and Iron Oxide Materials*, (ed. Shatokha, V.) 255 (IntechOpen, London, 2018).

[55] Persson, K. Materials Data on FeRhO_3_ (SG:221). *Materials Project,* (United States, 2016). https://materialsproject.org/materials/mp-973648/.

[56] Douglas BE, Ho SM (2006). Structure and chemistry of crystalline solids, 95.

[57] Klencsár, Z. *MossWinn.* https://www.mosswinn.com/.

[58] Sajti S, Tanczikó F, Deák L, Nagy DL, Bottyán L (2015). Angular dependence, blackness and polarization effects in integral conversion electron Mössbauer spectroscopy. Nucl. Instr. Methods B.

[59] Ahmad I (1995). Half-lives of isomeric states in ^57^Fe and ^83^Kr. Phys. Rev. C.

[60] Spiering H.,·*et al.* Line shape of ^57^Co sources exhibiting self absorption. *Hyp. Int.* **58**, 237 (2016).

[61] Patterson AL (1939). The Scherrer formula for X-ray particle size determination. Phys. Rev..

[62] Shirane G, Chen CW, Flinn PA, Nathans R (1963). Mössbauer study of hyperfine fields and isomer shifts in the Fe-Rh alloys. Phys. Rev..

[63] Alekseev IE (2012). Preparation of ^57^Co Mössbauer sources in a chromium matrix: Methodological aspects. Radiochemistry.

[64] Gruner ME, Hoffmann E, Entel P (2003). Instability of the rhodium magnetic moment as the origin of the metamagnetic phase transition in α–FeRh. Phys. Rev. B.

[65] Jekal S, Rhim SH, Hong SC, Son W, Shick AB (2015). Surface-termination-dependent magnetism and strong perpendicular magnetocrystalline anisotropy of an FeRh (001) thin film. Phys. Rev. B.

[66] Gu RY, Antropov VP (2005). Dominance of the spin-wave contribution to the magnetic phase transition in FeRh. Phys. Rev. B.

[67] Ziegler JF, Biersack JP, Littmark U (1985). The Stopping and Range of Ions in Solids.

[68] Ziegler, J. F., Biersack, J. P., Ziegler, M. D. *SRIM website.* https://www.srim.org/.

[69] Oshima R, Hori F, Kibata Y, Komatsu M, Kiritani M (2003). Defect structures and phase transitions of FeRh alloys deformed at high speed deformation. Mat. Sci. Eng..

[70] Rennhofer M (2010). Study of reorientation processes in L10-ordered FePt thin films. Intermetallics.

[71] Pászti F, Manuaba A, Hajdu C, Melo AA, da Silva MF (1990). Current measurement on MeV energy ion beams. Nucl. Instr. Methods B.

[72] Bottyán L (2013). GINA—A polarized neutron reflectometer at the Budapest Neutron Centre. Rev. Sci. Instrum..

[73] Bottyán L, Merkel DG, Nagy B, Major J (2012). Neutron Reflectometer with polarization option at the Budapest Neutron Centre. Neutron News.

