# Supplementary material for: Reversible control of magnetism in FeRh thin films
Source: Sci Rep. 2020 Aug 18;10:13923. doi: 10.1038/s41598-020-70899-x (PMC7435192; doi:10.1038/s41598-020-70899-x)
Supplement: Supplementary file 1 — Supplementary information [file 41598_2020_70899_MOESM1_ESM.docx]

Supplementary Information

# Reversible control of magnetism in FeRh thin films

Dániel G. Merkel^1,2*^, Attila Lengyel^1^, Dénes L. Nagy^1^, Attila Németh^1^, Zsolt E. Horváth^2^, Csilla Bogdán^1^, Maria A. Gracheva^3^, Gergő Hegedűs^1^, Szilárd Sajti^1^, György Z. Radnóczi^2^ & Edit Szilágyi^1^

^1^Wigner Research Centre for Physics, P.O.B. 49, 1525 Budapest, Hungary

^2^Centre for Energy Research, P.O.B. 49, 1525 Budapest, Hungary

^3^Laboratory of Nuclear Chemistry, Institute of Chemistry, ELTE Eötvös Loránd University, Pázmány Péter sétány 1/A, 1117 Budapest, Hungary

^*^Corresponding author’s email: [merkel.daniel@wigner.hu](file:///C:\Nagy\MUNKABAN\Mossba_lab_NDL\Projects\MBE_81001_FeRh\Paper\merkel.daniel@wigner.hu)

## Methodological approach of CEMS spectrum fitting

### General considerations

The basic methodological approach of the evaluation of the CEMS spectra was separating broadenings from the source and instrumental vibrations on one hand and those from the distribution of hyperfine interactions in the sample on the other hand. The source spectrum was described by a pseudo-Voigt function accounting for resonant self-absorption in the source ^[[1]](#endnote-1)^ and instrumental vibrations. Since thickness effects are negligible for perpendicular-incidence CEMS, in this approach, the widths of the resonant lines of the sample are no fit parameters but are set equal to the natural linewidth 0.0960 mm/s calculated from the 99.2 ns half-life of the 14.4 keV isomeric state of ^57^Fe ^[[2]](#endnote-2)^ unless some unresolved distribution of the hyperfine fields in the sample has to be accounted for. The experimental spectrum is then the convolution of the source and sample spectra.

All CEMS spectra of the sample taken in its different states (i.e. 1. *As-deposited*, 2. *Annealed*, 3. *Irradiated*, and 4. *Re-annealed*) were fitted simultaneously with constrains described later.

The components of all spectra fall in three main groups:

1. Magnetic (FM or AFM) components from the B2 phase.
2. Nonmagnetic (PM) contribution from the A1 phase.
3. Quadrupole doublet from the surface oxide layer.

In the following, the models applied for these groups will be described in more detail.

### Magnetic components of the B2 phase

Magnetic components of the B2 phase of Fe-Rh alloys were identified and classified by Shirane et al. in 1963 ^[[3]](#endnote-3)^. In case of a minor excess of Fe as compared with the stoichiometric Fe_50_Rh_50_ composition, most Fe atoms occupy their regular sites in the ordered B2 lattice (Fe I atoms), however the excess Fe atoms go to Rh sites (Fe II atoms). FM Fe I, AFM Fe I and Fe II atoms can be well separated on the basis of their hyperfine magnetic fields (see in more detail in the Discussion chapter of the main paper).

Since the late 1960-es, the widely accepted approach of describing hyperfine fields in dilute A2 (bcc) Fe alloys has been supposing that both the hyperfine magnetic field *H* and the isomer shift *δ* are the sum of contributions from nearest and next-nearest neighbours and that these contributions are proportional to the numbers of impurity atoms *n* and *m* in the first and second coordination spheres, respectively (cf. Ref. ^[[4]](#endnote-4)^ and references therein):

$H\left( n,m \right)=H_{0}+n\Delta H_{1}+m\Delta H_{2}$ (S1)

$\delta\left( n,m \right)=\delta_{0}+n\Delta\delta_{1}+m\Delta\delta_{2}$ (S2)

(cf. Eqs. (4) and (5) of Ref. [4]) while the quadrupole interaction was neglected. The simultaneous probability distribution of *n* and *m* is the product of two binomial distributions describing the probabilities of finding *n* and *m* impurities in the first and second coordination spheres of 8 and 6 sites, respectively in the bcc lattice at impurity concentration *c*:

$p\left( n,m,c \right)=\binom{8}{n}c^{n}\left( 1-c \right)^{8-n}\binom{6}{m}c^{m}\left( 1-c \right)^{6-m}$ (S3)

(cf. Eq. (3) of Ref. [4]).

Postulating that, in the case under discussion, the antisite Fe II atoms are randomly distributed in the B2 lattice and that their influence on the hyperfine interactions of the Fe I atoms can be described with the same technique (i.e. as if the Fe II atoms were dilute impurities in the stoichiometric B2 Fe_50_Rh_50_ lattice). Then, *c* = 0.02 corresponds to the composition Fe_51_Rh_49_, *c* = 0.04 to Fe_52_Rh_48_, etc.

In the present analysis, this model was further simplified by neglecting the influence of the second coordination sphere. Eqs. (S1–S3) are then replaced by

$H\left( n \right)=H_{0}+n\Delta H_{1}$ (S4)

$\delta\left( n \right)=\delta_{0}+n\Delta\delta_{1}$ (S5)

$p\left( n,c \right)=\binom{8}{n}c^{n}\left( 1-c \right)^{8-n}$ (S6)

This approximation is fully justified for the Fe I atoms, the second neighbours of which are always Fe I atoms since there is no reason to suppose that Rh atoms appear in a Rh-deficient alloy at Fe sites of the B2 lattice. Conversely, the antisite Fe II atoms occupy Rh sites so that for the same reason they have exactly 8 first Fe I neighbours. The distribution of their second neighbours (mostly Rh and some Fe II) could, admittedly, lead to a minor scatter of their hyperfine interactions. However, in terms of the above approximation, the hyperfine magnetic field *H*^*^ and the isomer shift *δ*^*^ of the Fe II atoms have sharp, well-defined values.

Adjusted parameters of the simultaneous fit of all CEMS spectra were *c*, *H*^*^, *δ*^*^, *H*_0_, *ΔH*_1_, *ρ*_0_ and *Δδ*_1_. Furthermore, the magnetic texture, i.e. the average direction of the hyperfine magnetic field relative to the sample was evaluated. Line intensities of magnetic sextets were described as 3:*a*:1:1:*a*:3, *a* being the measured texture parameter. The magnetic texture for all sextets in the same state of the sample was supposed to be the same. The values *a* = 4, 2 and 0 correspond to plane-parallel, random (or magic-angle, i.e. 54.7 ° to the plane normal) and plane-perpendicular orientation of the hyperfine magnetic field (and the magnetization), respectively. Since significant contributions from magnetic sextets appear only in the *Annealed* and *Re-annealed* states of the sample, for the minor magnetic contributions in the *As-deposited* and the *Irradiated* states the same *a* values were used as in the *Annealed* and *Re-annealed* states, respectively, with the implication that the observed change of *a* was due to the irradiation (see in more detail in the Discussion chapter of the main paper).

Although, in principle, *H*_0_, *ΔH*_1_, *δ*_0_ and *Δδ*_1_ could have two co-existing values corresponding to the FM and AFM states, only those consistent with the FM state were confirmed by the analysis of the spectra.

### Nonmagnetic contribution from the A1 phase

The PM A1 phase observed mainly in the *As-deposited* and the *Irradiated* states of the sample contributes basically with a single line to the spectra. However, this single line of the sample spectrum cannot be fitted with the same Lorentzian as in calibration, a fact certainly due to an unresolved distribution of the hyperfine interactions as a consequence of some defects. Therefore, in contrast to other components, the sample spectrum of the A1 phase was fitted with a pseudo-Voigt function of fixed Lorentzian width equal to the natural linewidth and a free Gaussian width. This means that the unresolved distribution of the hyperfine interaction was described by a Gaussian distribution. Accordingly, the A1 contribution has two hyperfine fit parameters: the isomer shift *δ*_A1_ and the Gaussian width of the distribution of the unresolved hyperfine interaction *σ*_A1_. Since the contribution of the A1 phase is significant only in the *As-deposited* and *Irradiated* states, the minute A1 fraction in the *Annealed* and *Re-annealed* states was fitted with the same values of *δ*_A1_ and *σ*_A1_ as those of the *Irradiated* state, with the implication that the observed change of these parameters was due to the first annealing of the *As-deposited* sample (for further details see the Discussion chapter of the main paper).

### Contribution of the surface oxide layer

The sample spectrum of the surface oxide layer was described by a symmetric quadrupole doublet of lines of natural width. Therefore, this component has two fitted hyperfine parameters: the isomer shift *δ*_ox_ and the quadrupole splitting *ΔE*_ox_.

### Relative fraction of the contributions

The respective percentile fractions of the B2 and A1 phases *r*_B2_ and *r*_A1_ were fit parameters free in all states of the sample. Supposing that the recoilless fractions of the B2 and A1 phases are the same, the relative fraction of the magnetic B2 phase within the multilayer is characterized by the derived quantity *ρ*_B2_ = *r*_B2_/(*r*_B2_/*r*_A1_).

The percentile fraction of the surface oxide contribution within the whole spectrum *r*_ox_ was a common fit parameter for all spectra. Even if the recoilless fraction of the oxide were the same as those of the B2 and A1 phases, its fitted value definitely overestimates the atomic fraction of the oxide in the sample due to the fact that the mean free path of conversion electrons in similar systems is not more than about 50 nm ^[[5]](#endnote-5)^. However, this fact has no influence either on the value of the hyperfine parameters or on the value of *ρ*_B2_.

Detailed fit results are summarized in Table S1.

| \| **Parameter** \| **As-deposited** \| **Annealed** \| **Irradiated** \| **Re-annealed** \| \| --- \| --- \| --- \| --- \| --- \| \| *c* \| 0.0208(2) \| 0.0208(2) \| 0.0208(2) \| 0.0208(2) \| \| *ρ*_B2_ \| 0.060(3) \| 1.000(1) \| 0.008(7) \| 0.999(3) \| \| *H*^*^ (T) \| 39.2(4) \| 39.2(4) \| 39.2(4) \| 39.2(4) \| \| *δ*^*^ (mm/s) \| 0.273(2) \| 0.273(2) \| 0.273(2) \| 0.273(2) \| \| *H*_0_ (T) \| 27.135(2) \| 27.135(2) \| 27.135(2) \| 27.135(2) \| \| *ΔH*_1_ (T) \| 1.980(9) \| 1.980(9) \| 1.980(9) \| 1.980(9) \| \| *δ*_0_ (mm/s) \| 0.0214(2) \| 0.0214(2) \| 0.0214(2) \| 0.0214(2) \| \| *Δd*_1_ (mm/s) \| 0.014(1) \| 0.014(1) \| 0.014(1) \| 0.014(1) \| \| *a* \| 3.25(1) \| 3.25(1) \| 2.32(2) \| 2.32(2) \| \| *δ*_A1_ (mm/s) \| 0.0462(3) \| 0.0462(3) \| 0.0565(7) \| 0.0565(7) \| \| *σ*_A1_ (mm/s) \| 0.163(1) \| 0.163(1) \| 0.207(2) \| 0.207(2) \| \| *δ*_ox_ (mm/s) \| 0.311(4) \| 0.311(4) \| 0.311(4) \| 0.311(4) \| \| *ΔE*_ox_ (mm/s) \| 0.715(7) \| 0.715(7) \| 0.715(7) \| 0.715(7) \| \| *r*_ox_ (%) \| 2.59(6) \| 2.59(6) \| 2.59(6) \| 2.59(6) \| |
| --- | --- | --- | --- | --- | --- | --- | --- | --- | --- | --- | --- | --- | --- | --- | --- | --- | --- | --- | --- | --- | --- | --- | --- | --- | --- | --- | --- | --- | --- | --- | --- | --- | --- | --- | --- | --- | --- | --- | --- | --- | --- | --- | --- | --- | --- | --- | --- | --- | --- | --- | --- | --- | --- | --- | --- | --- | --- | --- | --- | --- | --- | --- | --- | --- | --- | --- | --- | --- | --- | --- | --- | --- | --- | --- | --- |
| **Table S1.** ^57^Fe CEMS parameters of the as-deposited MgO(100)/ [^n^FeRh/^57^FeRh]_10_ sample and after the indicated treatment. The numbers in bracket mean the standard deviation in the last digits. |

## RHEED image of the as-deposited sample

In-situ reflection high-energy electron diffraction (RHEED) is a method routinely used for characterizing the quality of epitaxy in molecular beam epitaxy (MBE) machines during the growth process. The RHEED pattern is composed of two types of spots. Those arranged around a half circle originate from the dynamically reflected electrons from the high-quality epitaxial structure, while the others forming a rectangular pattern are the consequence of transmission diffraction through islands epitaxially grown on the surface.


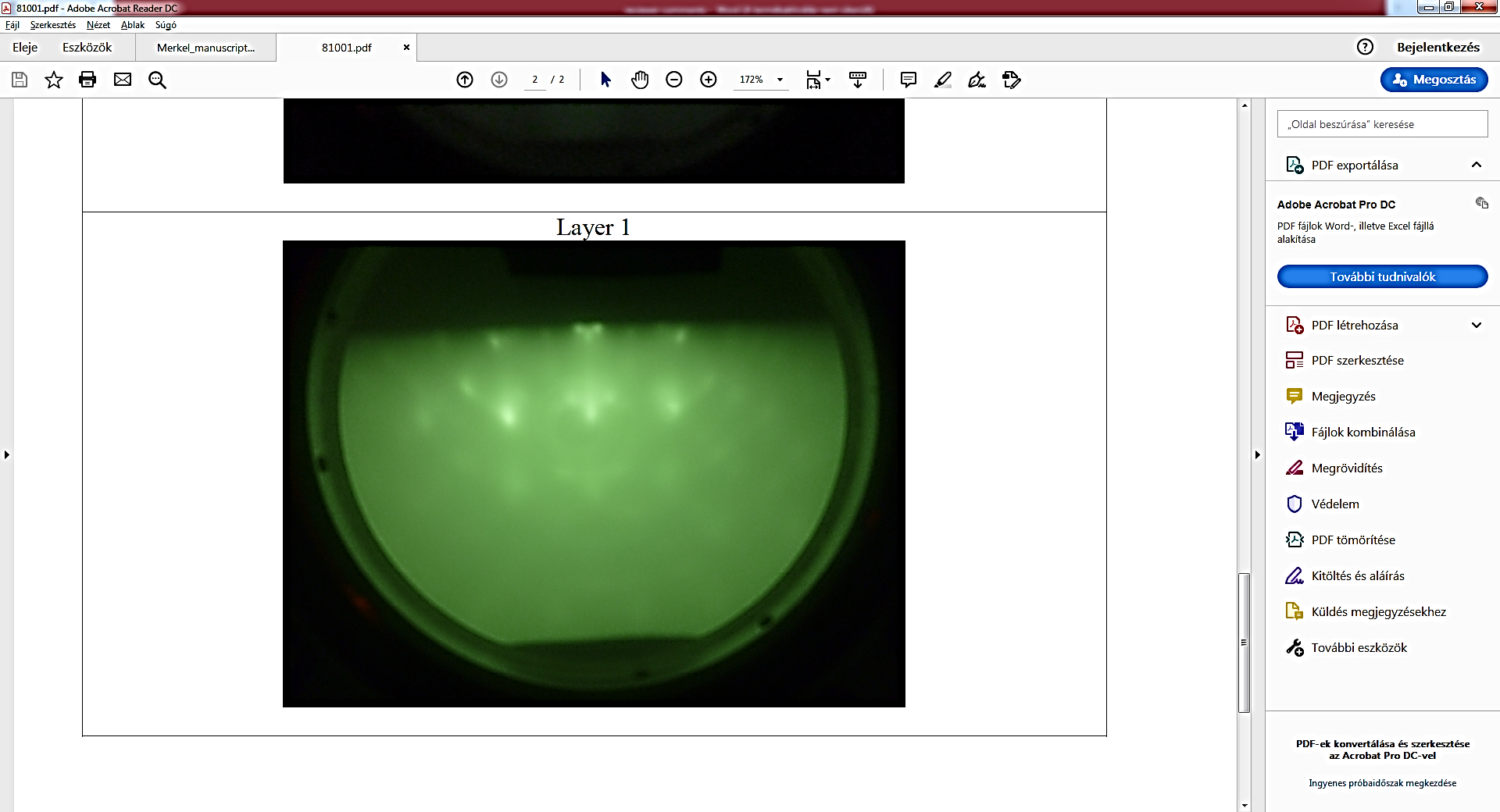


## References

1. Spiering, H. *et al.* Line shape of ^57^Co sources exhibiting self absorption. *Hyp. Int.* **58,** 237 (2016). [↑](#endnote-ref-1)
2. Ahmad, I. *et al.* Half-lives of isomeric states in ^57^Fe and ^83^Kr. *Phys. Rev. C* **52,** 2240 (1995). [↑](#endnote-ref-2)
3. Shirane, G., Chen, C. W., Flinn, P. A. & Nathans, R. Mössbauer study of hyperfine fields and isomer shifts in the Fe-Rh alloys. *Phys. Rev.* **131,** 183 (1963). [↑](#endnote-ref-3)
4. Vincze, I. & Campbell, I. A. Mössbauer measurements in iron based alloys with transition metals. *J. Phys. F: Metal Phys.* **3,** 647 (1973). [↑](#endnote-ref-4)
5. Sajti, Sz., Tanczikó, F., Deák, L., Nagy, D. L. & Bottyán, L. Angular dependence, blackness and polarization effects in integral conversion electron Mössbauer spectroscopy. *Nucl. Instr. Methods B* **342,** 62 (2015). [↑](#endnote-ref-5)
